# Book Review: *Making Thinking Visible*

**Published:** 2015-12-11

**Authors:** Kenneth Royal, Lizette Hardie

**Affiliations:** 1 Department of Clinical Sciences, North Carolina State University, United States

## Abstract

Recent trends in medical education emphasize the importance of producing well-rounded graduates who not only possess a sufficient fund of medical knowledge but who can think clearly and deeply. Unfortunately, many medical educators struggle with what exactly it means to “think” and do not know where to begin when asked to teach “thinking.” We believe the book *Making Thinking Visible*, though written for K-12, will be of great interest to medical educators. The book describes different types of thinking and presents more than 20 teaching routines that can help engage learners and improve learning to think.

## Introduction

Medical education is currently experiencing much change. Most health professions are shifting toward competency-based education. This movement has resulted in a great deal of research and discussion about both the quantity and nature of the skills today’s graduates should possess. In all instances, it is agreed that simply having a plentiful fund of medical knowledge is insufficient, as all learners need to be well-rounded with various skills, values and attitudes. Similarly, medical education assessment has also improved significantly, as researchers and educators have been searching for and developing new and improved ways to assess students using methods that go beyond knowledge examinations (that unfortunately often measure little more than memorization).

Despite these many advances and improvements, a large number of medical educators still struggle with some fundamental, albeit complex, questions such as what it means to “think.” Notwithstanding the increased interest in exploring new instructional techniques, many faculty do not know where to begin when faced with teaching the process of “thinking.” Because most medical educators have not formally studied education and pedagogy, we believe the book *Making Thinking Visible* may be of particular interest, as the authors identify different types of thinking and present more than 20 teaching routines that can help engage learners and improve the learning process for all involved. Giving students overt, specific, and intentional thinking routines gives them the tools to process complex concepts and problems.

## Organization of the Text

The text consists of three primary parts. Part One “Some Thinking about Thinking” offers two chapters that explore what it means to think. The authors state, “If we want to support students in learning, and we believe that learning is a product of thinking, then we need to be clear about what it is we are trying to support.”(p5) The authors challenge Bloom’s taxonomy and argue that while the categories are a starting point, the taxonomy is not particularly useful as thinking is neither sequential nor hierarchical. The authors further argue that we should not focus our efforts on identifying different types of thinking, but rather on quality of thinking within a particular type of thinking.

In addition to fine-tuning conceptualizations of thinking, the authors also address how assessment practices sometimes work against efforts to help students think. The authors confront the practice of the delivery paradigm in which education focuses on covering material and testing students on that material. Most educators will admit that not everything that is taught will be learned, but because a test score provides some evidence of learning most educators readily accept these scores as sufficient evidence. The authors argue that practice based on the delivery paradigm “robs the teacher of the ability to confront students’ misconceptions and design experiences to advance their understanding.”(p27) The authors spend the remainder of this chapter arguing why thinking should be made visible.

Part Two, “Using Thinking Routines to Making Thinking Visible,” consists of four chapters, with Chapter 3 providing an introduction to thinking routines. Chapters 4–6 present a series of specific teaching routines organized by purpose. For example, Chapter 4 focuses on routines for introducing and exploring ideas. Seven specific teaching routines are provided, each complete with a purpose, suggestions for selecting appropriate content, steps for implementing the routine, ideas for uses and variations, suggestions for assessment, tips based on the experiences of the authors with the routine, and an example of the routine in practice.

Part Three, “Bringing the Power of Visible Thinking to Life,” consists of Chapters 7–8. These chapters primarily place each of the individual routines into the larger context of classroom culture and emphasize how each activity should not be treated as a stand-alone pedagogical approach, but rather one technique in a larger effort to build a culture in which ideas can be discussed and thinking made visible. The authors also discuss the importance of teachers modeling many of these practices in professional learning communities and other working groups to help create and solidify cultures of thinking.

## Evaluation of the Text

The greatest limitation of the text is its focus on K-12 education. Despite the differing context, most of the content can be extended to higher education, including medical education. For example, the first teaching routine presented in the text is entitled “See-Think-Wonder.” The routine asks students to look at an image or object and asks “what do you see?” “what do you think is going on?” and “what does it make you wonder?” The example provided involves a high school history teacher presenting a black and white image of *The Temptation of St. Anthony* on a projector screen for students to inspect. Using the routine, she was able to get students to identify subtle clues in the image (e.g. “chicken feet” and a tail on the woman) that helped discern what the image was conveying. This type of routine may be particularly useful in not only medical arts and humanities courses, but also pathology, radiology and other related courses/settings. In another example, the authors present “Think-Puzzle-Explore.” The teacher selected the concept of time, then asked “What do you think you know about time?” followed by “What questions or puzzles do you have?” then “How can we explore these puzzles?” Imagine this technique applied to diabetes. Virtually all of the 21 teaching routines could be extended to medical education in some capacity, but will likely require some creativity on the part of teachers.

The book possesses a number of strengths, not the least of which is its excellent research-based foundation. It is very easy to read and should not present any problems for most medical educators. Teaching and learning theory aside, many faculty will likely find the text particularly helpful as its “cook book” style makes the information particularly practical and illustrates exactly how one could make thinking visible. We highly recommend that many other medical educators explore this very practical and informative text.
